# Piezo channels in tumors

**DOI:** 10.1007/s00432-026-06474-0

**Published:** 2026-04-11

**Authors:** Wenxin Zhang, Liangliang Dai, Honglei Shi

**Affiliations:** 1 Department of Urology, Wujin Hospital Affiliated with Jiangsu University, Changzhou, 213004 China; 2 Department of Urology, The Wujin Clinical College of Xuzhou Medical University, Changzhou, 213004 China; 3 Changzhou medical center of Nanjing Medical University, Changzhou, 213004 China

**Keywords:** Piezo, Tumor, Cancer

## Abstract

**Introduction:**

Malignant tumors currently pose a significant threat to global health. Tumor progression is jointly regulated by genetic mutations and the mechanical properties of the tumor microenvironment (TME), including increased tissue stiffness, elevated fluid pressure, and mechanical compression experienced by circulating tumor cells (CTCs) within microvessels. These mechanical signals are transmitted through mechanosensitive pathways, with the Piezo channel family (Piezo1/Piezo2) serving as a core mediator.With their propeller-like trimeric structure, Piezo channels sense membrane tension, mediate calcium influx, and activate downstream signaling pathways (e.g., MAPK, PI3K/AKT/mTOR, YAP/TAZ), thereby regulating tumor cell proliferation, migration, immune microenvironment remodeling, and cancer stem cell-like transformation. Its expression exhibits tissue specificity and correlates with tumor staging, invasiveness, and pro gnosis.

**Results:**

Piezo1 is upregulated in breast, esophageal, colorectal, glioma, and prostate cancers to promote tumor progression, while its downregulation in lung cancer enhances malignancy.Notably, the Piezo1 channel can be activated by mechanical compression in microcapillaries, subsequently promoting circulating tumor cells to acquire stem cell-like properties through calcium signaling pathways, thereby enhancing their metastatic potential. This discovery not only reveals the pivotal role of mechanical forces in tumor metastasis but also positions the Piezo channel as a promising biomarker for tumor diagnosis and prognostic assessment. Currently, targeted strategies for the Piezo channel—including small-molecule modulators and treatments based on piezoelectric materials—are gradually opening new avenues for precision cancer therapy, although issues such as tissue specificity of function and drug selectivity require further exploration.

**Conclusion:**

Overall, as a vital bridge linking mechanical signals in the tumor microenvironment to cellular biological behavior, Piezo channels hold significant importance for deepening our understanding of tumor mechanisms, developing novel biomarkers, and optimizing therapeutic strategies.

## Introduction

In recent years, malignant tumors have become one of the major factors threatening human health worldwide (Bray et al. 2024). According to statistics, the number of cancer deaths worldwide is projected to reach 18.5 million by 2050 (Bizuayehu et al. 2024).

The development and progression of cancer represent a complex pathological process involving the combined effects of genetic mutations and microenvironmental factors. Among these, the tumor microenvironment (TME) has been identified as a key driver of cancer progression. Within the TME, diverse cellular and non-cellular components—including blood vessels, immune cells, fibroblasts, endothelial cells, and the extracellular matrix (ECM)—interact synergistically to promote tumor growth and metastasis (Zhang et al. 2026, Ferrari et al. 2019). Compared to normal tissues, excessive deposition and cross-linking of the extracellular matrix (ECM) within the tumor microenvironment often leads to a significant increase in tissue stiffness. In some tumor tissues, stiffness can reach 10–100 times that of normal tissues (Chen et al. 2018). In addition to increased intravascular fluid pressure caused by abnormal angiogenesis, circulating tumor cells (CTCs) must overcome mechanical compression stresses of 20–50 kPa when traversing microcapillaries with diameters of 5–10 μm (Liang et al. 2024). The process by which mechanical signals (such as pressure, tension, shear force, and matrix stiffness) are converted into intracellular biochemical signals via specific ‘mechanoreceptors’ is termed mechanotransduction. This process has diverse biological implications in tumor progression, including regulating tumor cell motility, cytokine release, and other cellular behaviors; among these effects, it can significantly promote tumor invasion and metastasis under certain contexts (Bao et al. 2024, Felice and Alaimo 2020, Geng and Wang 2016). Mechanosensitive ion channels (MSCs) are a class of membrane proteins that include epithelial sodium channel protein (ENaC), the Piezo channel family, Trek subfamily proteins, transient receptor potential (TRP) family proteins, and the transmembrane protein 16 (TMEM16) superfamily (Jin et al. 2020). These channels form the core mechanism by which cells sense and transduce mechanical signals. They rapidly activate in response to mechanical stimuli on the cell membrane, regulating ion transport across the membrane (Cox et al. 2019, He et al. 2024, Coste et al. 2010, Wan et al. 2024). Acting on downstream effectors, they mediate diverse cellular responses including growth, migration, adhesion, and vesicular transport (Otero-Sobrino et al. 2023, Gu and Gu 2014).

The Piezo family comprises two subtypes, Piezo1 and Piezo2 (Coste et al. 2010), which are widely distributed throughout various tissues and organs in the human body, such as the cardiovascular, respiratory, gastrointestinal, urinary, and skeletal systems. They participate in multiple physiological processes, including tactile sensation, blood pressure regulation, and the control of cellular functions (Xiao 2020, Yu and Liao 2021, Savadipour et al. 2023, Shah et al. 2022). Within the tumor microenvironment, abnormal mechanical signals can activate Piezo channels. By mediating calcium ion influx, these channels trigger a cascade of downstream signaling pathways that regulate tumor cell proliferation, invasion, and migration. Concurrently, they influence immune cell function and angiogenesis, thereby fostering a pro-tumor microenvironment (Sheth and Esfandiari 2022). Previous studies have demonstrated that Piezo channels play a crucial role in the development and progression of various tumors, including gastric cancer (Yang et al. 2014), lung cancer (Huang et al. 2019), glioma (Yang et al. 2016), and breast cancer (Li et al. 2015). Furthermore, their expression levels are closely associated with tumor staging and prognosis, making them potential diagnostic markers and therapeutic targets.

This review summarizes the structure, mechanisms, physiological functions, and pathophysiology of Piezo channels in cancer. It explores their potential applications in tumor diagnosis and treatment, concluding with a summary of current research challenges and future directions. The aim is to provide a comprehensive reference for Piezo channel-related tumor research and clinical translation.

## Molecular structure and functional characteristics of the piezo channel

### Molecular structure

Piezo1 (originally designated Fam38A) was discovered in 2010 in a mouse neuroblastoma cell line. Subsequently, researchers identified its family protein Piezo2 (formerly Fam38B) through sequence homology alignment (Felice and Alaimo^ 2020^, Coste et al. 2010, Swain and Liddle 2023). In terms of structural composition, both Piezo1 and Piezo2 are macromolecular proteins. Specifically, Piezo1 comprises approximately 2521–2547 amino acid residues, while Piezo2 consists of approximately 2752–2822 amino acid residues. It is noteworthy that each subunit contains up to 38 transmembrane segments (TM), a number significantly higher than that of conventional ion channels (Zhao et al. 2018, Saotome et al. 2018). These monomers further assemble into homotrimers, forming functional channel complexes on the cell membrane with a total molecular weight of approximately 900 kDa. This trimer structure serves as the core unit enabling ion conduction in the Piezo channel (Shah et al. 2022).

**Fig. 1:**
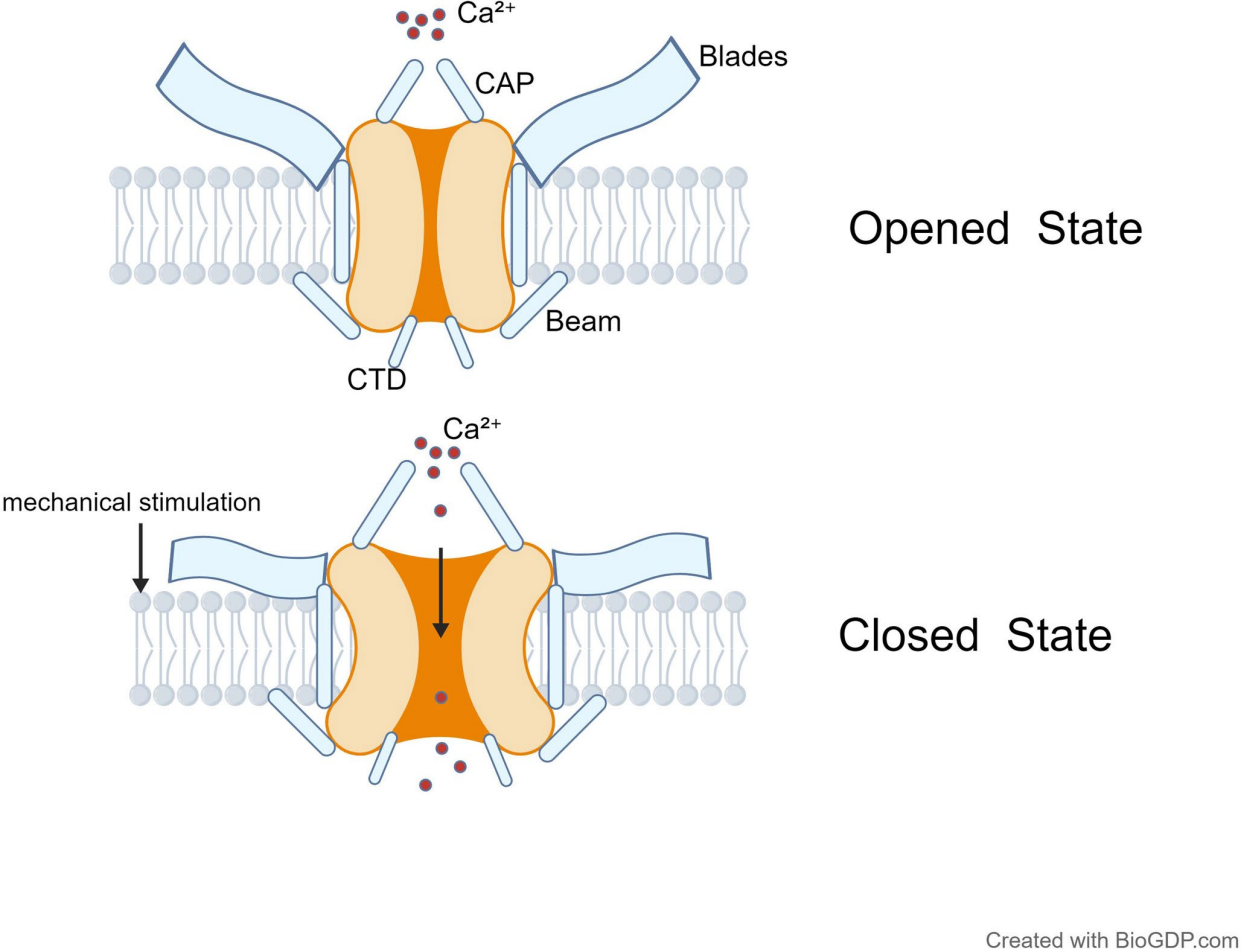
In the resting closed state, Piezo channels adopt a compact conformation with the central pore occluded. Upon mechanical stimulation, the peripheral blades and intracellular beam-like structures undergo reorientation to transmit mechanical force, causing pore dilation and opening. This mediates the influx of cations, primarily Ca^2+^, converting mechanical force into electrical/chemical signals. The CED (CAP) and CTD domains regulate force transmission and gating kinetics during this process

In terms of spatial structure, the piezo channel exhibits a unique three-bladed propeller-like conformation, with three blades arranged around the central ion-conducting pore, collectively forming an inverted bell-shaped overall structure (Zhao et al. 2018, Wang et al. 2019). Each leaflet comprises nine transmembrane helical units (THUs), with each THU containing four transmembrane (TM) helices that form a bent, non-planar conformation. This unique structure enables interaction with the lipid bilayer, allowing it to respond to changes in membrane tension or curvature (Hong et al. 2023). The blades are interconnected by intracellular beam-like structures approximately 90 Å in length. These beam-like components serve as intermediate carriers for mechanical force transmission, conveying mechanical signals detected by peripheral blades to the central pore region (Tang et al. 2023, He et al. 2023). Three C-terminal extracellular domains (CEDs) form a cap-like structure at the top of the channel, serving as a critical junction connecting the leaflets to the ion conduction pathway. Beyond playing a central role in establishing channel inactivation dynamics, it mediates the transmission of mechanical forces from the N-terminal to the C-terminal, thereby regulating pore opening and closing (Ludlow et al. 2025, Yang et al. 2025). The central ion-conducting pore module is the key region for Piezo channel ion permeability, primarily composed of the outer helical segment (OHS), inner helical segment (IHS), intracellular C-terminal domain (CTD), and CED, collectively forming a non-selective cation conduction pathway (Li et al. 2022). Among these, the central pore formed in the C-terminal region plays a pivotal role in determining channel conductance and ion selectivity (Fig. 1) (Wan et al. 2024, Jiang et al. 2025). Although both Piezo1 and Piezo2 are non-selective cation channels permeable to multiple cations including Ca^2+^, K^+^, Na^+^, and Mg^2+^, they exhibit the highest selectivity for Ca^2+^(Cha and Thibeault 2025, Li et al. 2023). However, subtle structural differences exist in their central pores. —Piezo2’s central pore is narrower than that of Piezo1, and its outer cap more tightly envelops the central pore. This structural difference may correlate with their functional specificity (He et al. 2024). Additionally, the hydrophobic gate within the inner helix (e.g., Piezo1 L2475/V2476) mediates rapid inactivation, while positively charged residues (e.g., K2479) participate in voltage-dependent regulation of the inactivation rate (Zheng et al. 2019).

The sequence homology between Piezo1 and Piezo2 is approximately 42–47% (Swain and Liddle 2023). Both exhibit high similarity in overall topological structure (38-TM), core functional module composition (mechanosensing module, transduction module, ion-conducting pore module), and key structural domain features, suggesting that their mechanical regulation mechanisms also tend to be similar (Hong et al. 2023). However, beyond differences in the central pore and outer cap structures, Piezo2 exhibits extensive alternative splicing, yielding multiple isoforms with distinct functional characteristics. These isoforms vary in inactivation rates, ion permeability, and intracellular calcium regulation, enabling adaptation to the functional demands of different cell types and tissues (Wan et al. 2024, Bais and Giudice 2025). Advances in cryo-electron microscopy (cryo-EM) technology have yielded significant breakthroughs in resolving the structure of the Piezo channel. These studies not only elucidated its overall trimeric propeller-like architecture but also revealed key structural features such as leaflet bending, intracellular beam connections, and hydrophobic gate positioning (Mazal et al. 2025, Zhang et al. 2025). Collectively, these structural elements determine the unique mechanical sensitivity of the Piezo channel, enabling it to convert mechanical stimuli into ionic signals. This provides the structural foundation for its functional role in both physiological and pathological processes.

### Core functions and signal transduction mechanisms

As key mediators of mechanical signal transduction in mammals, the core function of Piezo channels lies in sensing and converting mechanical stimuli, thereby participating in diverse physiological and pathological processes by regulating downstream signaling pathways. Although Piezo1 and Piezo2 exhibit differences in expression patterns and consequently display functional specificity, they still share partially conserved signaling mechanisms (Wang et al. 2025, Nottmeier et al. 2023).

Piezo1 is widely expressed in non-sensory tissues, particularly enriched in organs and cells influenced by fluid pressure and matrix stiffness, such as the cardiovascular system, intestines, kidneys, and mesenchymal stem cells. It plays a crucial role in mechanical force sensing, regulation of internal homeostasis, and tissue development (Itson-Zoske et al. 2023, Alvarez-Lorenzo et al. 2023). Piezo1 may induce relaxation of airway smooth muscle cells through calcium signaling and activation of large-conductance calcium-activated potassium channels (BK), offering a novel therapeutic approach for airway constrictive diseases such as asthma (Luo et al. 2025). In the cardiovascular system, Piezo1 regulates vascular endothelial cell polarization and contraction by sensing fluid shear stress generated by blood flow. Knockout of this gene leads to embryonic vascular developmental defects and impaired angiogenesis, ultimately resulting in embryonic lethality (Carrillo-Garcia et al. 2021, Fang et al. 2022). During skeletal development, Piezo1 serves as a critical mechanical sensor in bone marrow mesenchymal stromal cells and osteoblasts. Its absence leads to impaired osteogenic differentiation and spontaneous fractures in newborn mice (He et al. 2024). Recent studies confirm that Piezo1 is functionally expressed in cholinergic enteric neurons, where it regulates gastrointestinal motility by sensing mechanical stress and maintains intestinal immune homeostasis by limiting abnormal inflammatory responses, thereby providing a novel potential therapeutic target for inflammatory bowel disease (Zhang et al. 2025). Additionally, Piezo1 participates in tumorigenesis and immune regulation, with its overexpression negatively correlated with overall survival in cancer patients. It can also specifically inhibit Treg cells to alleviate experimental autoimmune neuritis (He et al. 2024).

Piezo2 is primarily localized in sensory tissues such as dorsal root ganglia and Merkel cells (Yang et al. 2022), with its core function focused on perceiving and transmitting low-threshold mechanical stimuli. It serves as a key receptor mediating light touch, proprioception, and certain visceral sensations (Li et al. 2023, Foote et al. 2022). Previous studies have demonstrated that mechanical stimulation activates Piezo2 in Merkel cells, generating force-dependent currents that subsequently induce downstream action potentials. Knockout mice lacking the Piezo2 gene exhibit severe tactile loss, impaired motor coordination, and abnormal limb position perception, while other sensory functions remain unaffected. This confirms Piezo2’s role as a tactile and proprioceptive receptor at both functional and behavioral levels (Marasco et al. 2023). Subsequent studies further revealed that Piezo2 plays a crucial role in mechanical hypersensitivity, airway dilation, and pulmonary ventilation regulation. Its expression levels serve as an indicator for assessing tissue sensitivity to mild mechanical stimuli following injury or inflammation (Chen et al. 2024, Nonomura et al. 2017, Schappe et al. 2024). Notably, Piezo2 shares a similar cation permeability order with Piezo1 (Ca^2+^> K^+^> Na^+^> Mg^2+^), but possesses a unique 10 nm-deep nanodome structure with dual constriction sites in its channel. This confers significantly higher sensitivity to low-threshold mechanical stimuli compared to Piezo1. This structural feature provides the basis for its sensory functional specificity (Fang et al. 2021, Xu et al. 2024, Wang et al. 2024).

The signal transduction mechanism of piezo channels centers on the conversion of mechanical force into bioelectric/biochemical signals (Li et al. 2023, Verkest et al. 2022). It relies on the channels’ intrinsic structural properties and interactions with the cellular microenvironment to form a multi-pathway regulatory network, where Ca^2+^ influx serves as a pivotal hub for signal transduction (Xiong et al. 2022).

Channel activation follows a dual “force-lipid” and “force-fiber” model: the former relies on membrane surface tension to induce lipid rearrangement, driving channel conformational changes via hydrophobic mismatch; Piezo1 can be activated at membrane tensions as low as 3.4 mN/m. The latter regulates channel conformation through direct traction exerted by cytoskeletal and extracellular matrix (ECM) proteins. Myosin II-associated traction modulates spontaneous Ca^2+^ transients in Piezo1, establishing a feedback loop of “mechanical signal → channel activation → cytoskeletal reorganization” (He et al. 2023, Zeitzschel and Lechner 2024). ECM proteins exert significant regulatory effects on the sensitivity of Piezo channel activation. In the absence of ECM, Piezo1 exhibits minimal responsiveness to mechanical stretching, whereas the mechanically connected network formed by the matrix gel substantially enhances its sensitivity and promotes channel activation (Roeterink et al. 2024, Mierke 2024). Additionally, membrane cholesterol content modulates the activation latency and inactivation rate of Piezo1, and treatment with methyl-β-cyclodextrin (MBCD) significantly alters its function, suggesting that the lipid microenvironment is a key factor in regulating channel activity (Nourse et al. 2022, Short 2020, Wenqiang et al. 2024). Upon activation by mechanical stimulation, the Piezo channel functions as a non-selective cation channel to mediate efficient Ca^2+^ influx. Acting as a second messenger, Ca^2+^ activates multiple downstream signaling pathways, thereby regulating cellular functions (Huang et al. 2023). Key downstream pathways include: the MAPK pathway, which regulates mesenchymal stem cell migration by activating PYK2 and MEK/ERK signaling (Cui et al. 2023, Mousawi et al. 2020); the PI3K/AKT/mTOR pathway, which promotes Akt phosphorylation via Ca^2+^ influx and participates in cell proliferation and survival regulation (Luo et al. 2023, Cheah et al. 2023) the YAP/TAZ pathway, which influences cell growth and metastasis by regulating Hippo pathway activity (Lee et al. 2022); the Wnt/β-catenin pathway, promoting cell proliferation and osteogenic differentiation (Kumar et al.^ 2025^ , Wang et al. 2025); and simultaneously activates calpain, NF-κB, and Notch pathways, participating in apoptosis, senescence, and differentiation regulation (Soydemir et al. 2019, Kondoh et al. 2021, Hilscher et al. 2019). In the central nervous system, Piezo1-mediated Ca^2+^ signaling also participates in processes such as brain development, synaptic function, axonal regeneration, and cerebral vascular remodeling. Its functional abnormalities are closely associated with spinal cord injury, stroke, and neurodegenerative diseases (Velasco-Estevez et al. 2018, Zheng et al. 2023, Xu et al. 2024). It is worth emphasizing that Piezo channel signaling exhibits rapidity and specificity, converting mechanical stimuli into biological signals within milliseconds without requiring additional proteins or second messengers. Its mechanical sensitivity stems from intrinsic properties, making it an efficient signaling hub in mechanotransduction (Cox et al. 2019, Audero et al. 2022)（Fig. 2）. 

## Piezo channel expression in different tumors

**Fig. 2:**
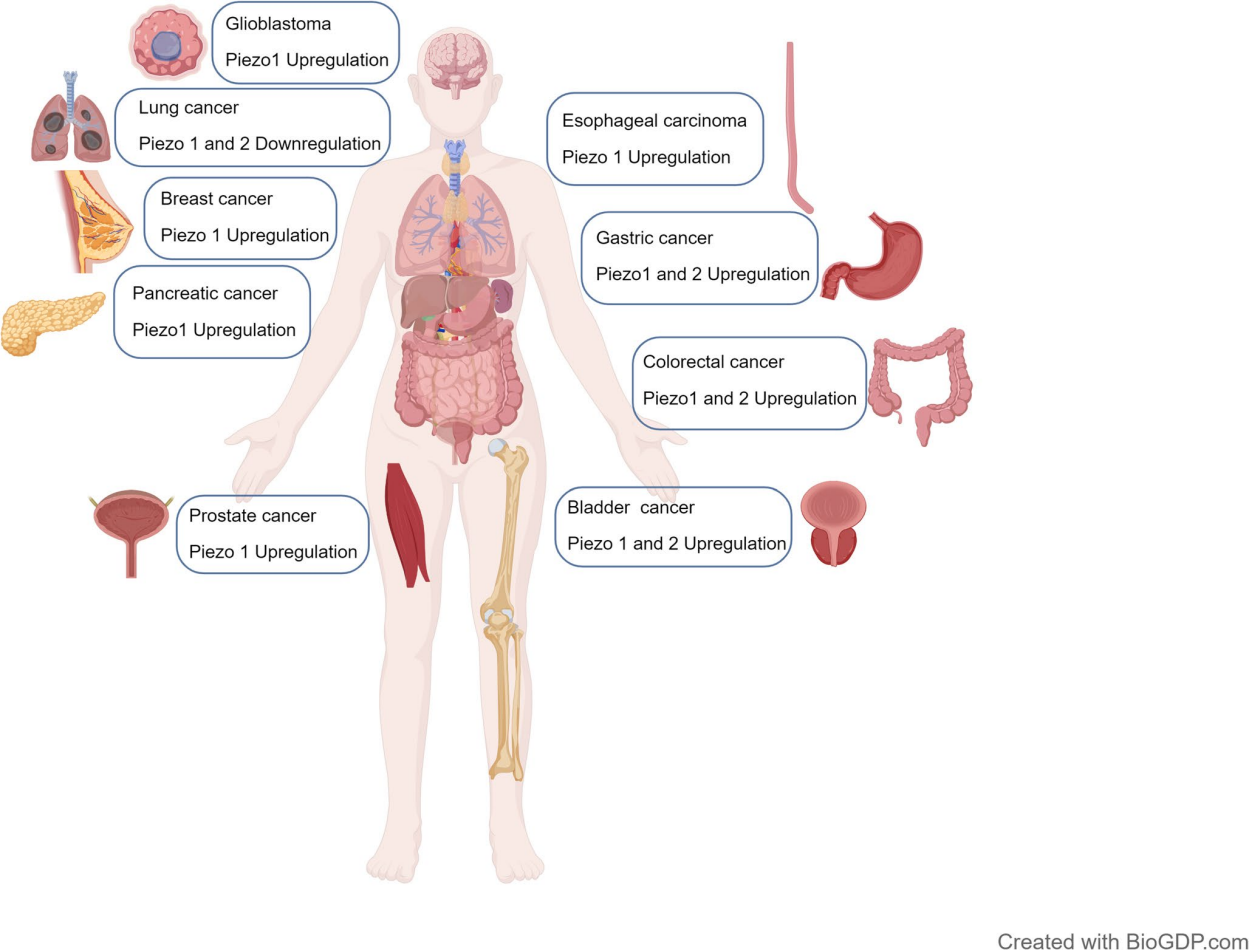
Expression of Piezo channels in malignant tumors of various systems (Jiang et al. 2025)

### Tumors of the digestive system

Piezo1 and Piezo2 exhibit subtype-specific expression patterns across various digestive system tumors. Their abnormal activation or dysregulated expression mediates mechanical signal transduction, immune microenvironment remodeling, and neuro-tumor interactions, In esophageal squamous cell carcinoma, Piezo1 exhibits significantly elevated expression levels positively correlated with tumor differentiation grade and lymph node metastasis rate. Patients with high Piezo1 expression demonstrated a 32% reduction in 5-year disease-free survival compared to those with low expression. Further mechanistic studies revealed that cellular crowding pressure generated during esophageal squamous cell carcinoma proliferation specifically activates Piezo1. By mediating Ca^2+^ influx, it initiates downstream signaling cascades that provide the driving force for cancer cells to breach the basement membrane and invade (Swain and Liddle 2023, Wang et al. 2025). In colorectal cancer, differential expression of Piezo1 correlates with tumor location, with significantly higher upregulation observed in left-sided colorectal cancers compared to right-sided tumors. This expression disparity is closely linked to the heterogeneity of mechanical stimulation from intestinal contents, stemming from anatomical and physiological differences between the left and right colon: The left colon has a relatively narrow lumen, leading to slow movement of intestinal contents (primarily formed stool). This subjects the intestinal wall to sustained high-intensity mechanical shear forces and compressive stresses, thereby driving epithelial-mesenchymal transition (EMT). In contrast, the right colon has a wider lumen where semi-liquid or liquid stool moves rapidly, generating significantly lower mechanical stimulation intensity than the left colon (Morales-Soto and Smith-Edwards 2025). This explains why Piezo1 is significantly more highly expressed in left-sided colorectal cancers. In vitro experiments confirm that Piezo1 knockdown reduces colorectal cancer cell migration by 47% while inhibiting lung metastasis formation in nude mouse xenograft models. (Yu and Liao 2021, Yang et al. 2022, Greenlee et al. 2022, Budinská et al. 2023). Piezo2 is highly expressed in colorectal cancer tissues, and its knockout inhibits the proliferation, migration, and invasion of SW480 cells (Shang et al. 2023). The role of Piezo channels in gastric cancer exhibits subtype differentiation characteristics, with Piezo2 primarily concentrated in tumor tissues at the gastroesophageal junction and gastric antrum. In patients with tumors ≥ 2 cm in diameter accompanied by refractory diaphragmatic spasm, Piezo2 positive expression reached 89%. Expression intensity correlated closely with tumor invasion depth and nerve involvement, suggesting Piezo2 as a potential biological marker for early gastric cancer warning and disease assessment (Liang et al. 2025, Zhang et al. 2024, Liang and Da 2023). Piezo1 is upregulated in diffuse gastric cancer, where it participates in constructing the inflammatory microenvironment by regulating the polarization direction of tumor-associated macrophages, thereby promoting peritoneal metastasis of cancer cells. Knockout of Piezo1 inhibits cell proliferation and invasion (Wang et al. 2021, Zhang et al. 2018). In pancreatic cancer, Piezo1 expression exhibits cellular specificity, being highly enriched in myeloid cells within the tumor microenvironment while showing lower expression levels in pancreatic cancer cells. The Piezo1 antagonist GsMTx4 and arachidonic acid demonstrate synergistic antitumor effects with oxaliplatin, offering a novel combination therapy strategy for HCC (Zhu et al. 2022).

### Central nervous system tumors

Glioblastoma, the most common primary brain tumor, is characterized by high invasiveness and poor prognosis. Abnormally elevated tissue stiffness and high intratumoral pressure within the tumor microenvironment represent key pathological features. Piezo1 is highly expressed in glioblastoma cells, with its expression levels positively correlated to tumor malignancy. It serves as a marker for assessing glioblastoma malignancy and prognosis (Chen et al. 2018). The mechanism of Piezo1 in gliomas is closely linked to the unique characteristics of the central nervous system: on one hand, glioma cells perceive mechanical stimuli in the tumor microenvironment through Piezo1, activating Ca^2+^ signaling pathways to promote cell proliferation and invasion while regulating the maintenance of tumor stem cell properties, leading to tumor recurrence and drug resistance; On the other hand, Piezo1 disrupts the homeostasis of the central nervous system microenvironment by modulating interactions between glial cells and neurons, thereby further promoting tumor progression (Chen et al. 2022). Due to the presence of the blood-brain barrier, traditional therapeutic approaches have limited efficacy. The emergence of piezoelectric nanomaterials offers a novel strategy for glioma treatment—by activating piezoelectric nanomaterials with ultrasound to generate localized electric fields, the activity of Piezo channels can be modulated. This approach enables antitumor effects while avoiding systemic side effects, demonstrating potential clinical application value (Wu et al. 2025).

### Breast cancer

Piezo1 is highly expressed in breast cancer, with its expression levels closely correlated with tumor invasiveness, clinical staging, and poor prognosis, making it a key oncogenic factor driving malignant progression of breast cancer (Felice and Alaimo 2020). Mechanical force signals regulate multiple downstream signaling pathways by activating Piezo1-mediated calcium influx, thereby promoting breast cancer cell invasion, migration, and restricted migration capabilities. Research indicates that abnormally elevated matrix stiffness and compressive forces within breast cancer tissues activate Piezo1 channels, leading to increased intracellular calcium ion concentrations. This, in turn, induces activation of the Src and ERK signaling pathways, promoting the formation of invasive pseudopodia. Invadopodia, as critical structures enabling tumor cells to degrade the extracellular matrix and achieve invasive migration, rely on actin cytoskeletal reorganization for their formation. Piezo1-mediated calcium signaling accelerates invadopodia formation and functional activation by regulating actin polymerization and depolymerization, thereby enhancing breast cancer cells’ extracellular matrix degradation capacity and invasive potential (Lopez-Cavestany et al. 2023). Additionally, Piezo1 can influence the interaction between breast cancer cells and their surrounding microenvironment by regulating the expression of cell adhesion molecules and cellular contractility, thereby promoting restricted migration within dense matrices—a process closely linked to local tumor invasion and distant metastasis (Tang et al. 2025). For therapeutic applications, a research team from Harbin Engineering University developed oxygen-vacancy-rich BiO_2−X_ nanosheets that utilize piezoelectric catalytic mechanisms for targeted breast cancer treatment. Under low-frequency ultrasound, these nanosheets activate piezoelectric effects, generating an internal electric field that catalyzes reactive oxygen species (ROS) production to induce tumor cell apoptosis. With negligible toxicity to normal tissues, this approach offers a novel strategy for precision breast cancer therapy and highlights the therapeutic potential of piezoelectric materials in oncology (Huang et al. 2024).

### Lung cancer

Unlike most tumors, Piezo1/2 exhibits unique expression and functional patterns in lung cancer. Its downregulation promotes lung cancer cell migration and anchorage-independent growth, while overexpression correlates with better prognosis in lung adenocarcinoma patients, suggesting a potential tumor-suppressing role for Piezo channels in lung cancer. This functional discrepancy may relate to the tissue origin of lung cancer, the mechanical characteristics of the tumor microenvironment, and the specific activation of downstream signaling pathways (Huang et al. 2019). Research on the precise regulatory mechanisms of Piezo channels in lung cancer remains limited; it is speculated that they may exert effects by modulating apoptosis, the cell cycle, or the epithelial-mesenchymal transition (EMT) process.

First, the lungs continuously undergo mechanical stretching and relaxation during respiration, creating a dynamic mechanical microenvironment with fluctuating tension. Unlike solid tumor tissues (such as breast or prostate) that primarily endure static mechanical stress, lung tissue remains in a state of periodic mechanical stretching. This characteristic directly regulates the structural conformation and functional activity of Piezo1/2 (Gong et al. 2021). In the lungs, the cyclic mechanical stretching induced by respiration may trigger structural rearrangements in Piezo1/2, modulating channel gating speed and consequently altering downstream signaling patterns. Under cyclic stretching, Piezo1/2 channels in lung cancer cells may exhibit slower inactivation rates, thereby attenuating oncogenic signaling and enhancing tumor-suppressive effects (Huang et al. 2019).

Secondly, lung tissue is rich in endothelial cells, smooth muscle cells, and immune cells. The interactions between these cells and lung cancer cells form a unique signaling network that jointly regulates the function of Piezo1/2. For example, in high-flow-induced pulmonary arterial hypertension, Piezo1 modulates pulmonary arterial smooth muscle cell (PASMC) proliferation and pulmonary arterial endothelial cell (PAEC) dysfunction through distinct signaling pathways: In PASMCs, Piezo1 upregulation activates the AKT/mTOR signaling pathway to promote cell proliferation. Conversely (Chen et al. 2022), in PAECs, it is regulated by the NF-κB p65 (RELA) transcription factor and participates in pulmonary inflammatory responses (Arenas et al. 2023). In lung cancer, abnormal expression of Piezo1/2 may disrupt this cell-specific signaling balance, shifting its function from oncogenic to tumor-suppressive.

Third, lung cancer primarily originates from respiratory epithelial cells, which are long-term adaptors to the pulmonary microenvironment (such as mechanical tension, oxygen concentration, and signaling molecules). Their intrinsic signaling pathways differ from those of other epithelial cells. As mechanical sensors, Piezo1/2 interact with signaling molecules unique to pulmonary epithelial cells, thereby generating distinct functional outputs. Piezo1 regulates the activation and fate of Postn+ myofibroblasts (Xu et al. 2025); while in epithelial-derived lung cancer cells, the interaction between Piezo1/2 and Postn may inhibit epithelial-mesenchymal transition (EMT), thereby exerting tumor-suppressing effects (Huang et al. 2019). Current research on the precise regulatory mechanisms of Piezo channels in lung cancer remains limited. It is speculated that Piezo1/2 may exert tumor-suppressing effects by modulating apoptosis, cell cycle, or EMT processes.

### Tumors of the urinary system

The expression levels of Piezo were significantly elevated in bladder cancer tissues, and their expression showed a positive correlation with tumor staging and grading, suggesting that Piezo channels may be involved in the malignant progression of bladder cancer. However, current research on the specific functions and mechanisms of Piezo channels in bladder cancer remains limited. It is speculated that they may activate calcium signaling pathways by responding to changes in intravesical fluid pressure, thereby promoting the proliferation, migration, and angiogenesis of bladder cancer cells. Furthermore, abnormal expression of the Piezo channel may also be associated with bladder cancer recurrence and drug resistance. Further research is needed to confirm its feasibility as a diagnostic biomarker and therapeutic target for bladder cancer (Etem et al. 2018). In prostate cancer, high intratumoral pressure activates Piezo1 channels, promoting proliferation and migration of prostate cancer cells via the Ca^2+^-Akt/mTOR signaling pathway. As a key pathway regulating cell growth, metabolism, and survival, abnormal activation of the Akt/mTOR pathway is a crucial mechanism in prostate cancer progression. Piezo1-mediated mechanical force signaling drives malignant proliferation by activating this pathway. Downregulating Piezo1 expression or blocking its activity significantly inhibits the proliferation and migration capabilities of prostate cancer cells, offering a novel direction for targeted therapy against prostate cancer (Han et al. 2019).

## The role of the piezo channel in tumor immune regulation

### Microcapillary squeeze-induced tumor cell stem cell-like transformation

Cancer cells circulating in the bloodstream must traverse microcapillaries with diameters as small as 5–10 μm, enduring immense deformation stress and mechanical compression. While conventional wisdom holds that such mechanical stress causes most cancer cells to die, recent research reveals that certain cancer cells can perceive and respond to this mechanical stimulus through the Piezo1-mediated signaling pathway. This triggers phenotypic reprogramming, transforming them into a tumor stem cell-like state with enhanced invasiveness and metastatic potential. Transcriptomic analysis revealed significant upregulation of multiple metastasis-associated genes (e.g., THY1, SUCNR1, COL1A2, COL3A1) in compressed cancer cells. Pathways including metabolic reprogramming, TGF*β* signaling, angiogenesis, and calcium signaling were activated, while cell cycle-related pathways were suppressed. At the protein level, compressed cancer cells exhibited marked upregulation of tumor stem cell markers such as CD44, CD271, ABCB5, and PRDM14 were significantly upregulated in squeezed cancer cells, which also exhibited enhanced tumor spheroid formation in serum-free culture, indicating stronger self-renewal and tumor initiation capabilities. In vivo experiments further confirmed that cancer cells squeezed through microcapillaries, when injected into the tail vein or intracardially, formed a greater number of pulmonary metastatic nodules and demonstrated stronger systemic metastatic potential, with significantly reduced survival in mice receiving these cells (Zhang et al. 2024).

### Piezo1-mediated calcium influx is the core mechanism for transferring phenotypic activation

In-depth mechanistic studies reveal that Piezo1 serves as the key “master switch” for microcapillary compression-induced tumor cell stem cell-like transformation. Mechanical compression induces Piezo1 to aggregate and activate on the cell membrane, mediating rapid calcium influx. This calcium influx is essential for subsequent chromatin remodeling, gene expression reprogramming, and activation of stem cell-like phenotypes. Experiments confirm that treating cancer cells with the Piezo1 agonist Yoda1 induces upregulation of stem cell markers and enhances tumor spheroid formation even without mechanical compression (Silvani et al. 2025). Conversely, mechanical compression-induced stem cell phenotypes were completely abolished upon Piezo1 knockout using antagonists GsMTx4, ruthenium red, or CRISPR technology. Compression experiments conducted in calcium-free medium similarly failed to induce stem cell phenotypes, conclusively validating the central role of Piezo1-mediated calcium influx in this process (Malko et al. 2023, Jäntti et al. 2022, Hu et al. 2023).

## The potential applications of piezo channels in tumor diagnosis and treatment

### As a biomarker for tumor diagnosis and prognosis assessment

Abnormal expression of the Piezo channel is closely associated with the clinical pathological characteristics and prognosis of various tumors, making it a promising novel biomarker for tumor diagnosis, classification, and prognostic assessment. In breast cancer (Li et al. 2015), gastric cancer (Yang et al. 2014, Zhang et al. 2018), colorectal cancer (Shang et al. 2023), glioma (Yang et al. 2016), and prostate cancer (Han et al. 2019), elevated Piezo1 expression correlates positively with advanced tumor staging, increased invasive and metastatic potential, and poor prognosis. It may serve as a potential indicator for assessing the malignancy and prognosis of these tumors. For example, the expression level of Piezo1 in gliomas correlates with tumor malignancy grade and peritumoral edema severity, aiding in glioma grading diagnosis and prognosis assessment (Yang et al. 2016); in laryngeal cancer, hypermethylation of the Piezo2 promoter serves as a risk factor for laryngeal cancer development and a prognostic indicator (Liouta et al. 2023). Furthermore, expression levels of downstream signaling molecules of Piezo channels (such as GRHL3, RNF114, and YAP) can serve as biomarkers for predicting the efficacy of tumor immunotherapy. For instance, tumors with high expression of GRHL3 or RNF114 exhibit poor response to PD-1 inhibitor treatment, making them negative predictors of immunotherapy efficacy. This helps identify suitable patient populations for immunotherapy, enabling precision medicine (Pang et al. 2024). Future large-scale clinical studies are needed to validate the specificity and sensitivity of Piezo channels and their downstream molecules as biomarkers, establish standardized detection methods, and advance their application in clinical diagnosis.

### As a new target for tumor-targeted therapy

Given the pivotal role of Piezo channels in tumor progression and immune regulation, targeting these channels has emerged as a novel therapeutic strategy for cancer treatment. This approach encompasses several directions, including small-molecule targeted therapy, combination immunotherapy, and piezoelectric material-mediated targeted therapy.

In the field of small-molecule targeted therapy, multiple specific modulators of Piezo1 have been identified, providing tools for targeted treatment. Piezo1 antagonists (such as GsMTx4 and ruthenium red) inhibit tumor cell proliferation, migration, invasion, and metastasis by blocking Piezo1 activity, while simultaneously enhancing CTL antitumor activity. These agents demonstrate therapeutic potential across various tumor models (Dela Paz and Frangos 2018). However, existing small-molecule drugs still face challenges such as insufficient specificity, poor in vivo stability, and limited delivery routes. Future efforts should focus on developing more potent, highly specific, and bioavailable Piezo channel modulators to advance their progression into clinical research.

Piezoelectric-mediated targeted therapy has emerged as a novel therapeutic strategy in recent years. By leveraging the piezoelectric effect generated in piezoelectric materials under external stimuli (such as ultrasound), it delivers electrical signals or reactive oxygen species to kill tumor cells, offering advantages of high specificity and safety. Newly developed BiO₂₋ₓ nanosheets rich in oxygen vacancies can generate an internal electric field under low-frequency ultrasound, catalyzing reactive oxygen species production to achieve in situ tumor clearance without significant toxicity to normal tissues. This offers a novel approach for precision treatment of tumors like breast cancer (Kang et al. 2021). The therapeutic efficacy of such piezoelectric materials may relate to regulating the activity of Piezo channels in tumor cells. Future research should further explore the interaction mechanisms between piezoelectric materials and Piezo channels, optimize material design, and enhance therapeutic outcomes.

## Conclusion

### Summary of findings

This review summarizes the structure, function, and mechanisms of Piezo channels (Piezo1 and Piezo2) in tumor progression. As key mechanosensitive ion channels, they convert mechanical stimuli from the tumor microenvironment into intracellular signals via calcium influx, thereby regulating tumor cell proliferation, invasion, and immune microenvironment remodeling. Piezo channels exhibit subtype-specific expression across different tumors, which is closely correlated with tumor staging and prognosis, indicating their potential as diagnostic biomarkers. Unlike previous studies (Felice and Alaimo 2020, Karska et al. 2023), we also describe a variety of emerging novel therapeutic strategies targeting Piezo channels, including small-molecule modulators and cutting-edge piezoelectric materials (e.g., BiO_2−X_ nanosheets). Such materials enable non-invasive, ultrasound-activated precision therapy for in situ tumor clearance, representing a promising direction for cancer treatment.

### Future perspectives

Despite progress, precise regulatory mechanisms of Piezo channels across different tumor types remain unresolved. Additionally, the clinical translation of Piezo channel-targeted strategies faces specific hurdles mentioned earlier in the text, such as insufficient drug specificity of small-molecule modulators, limited in vivo stability of therapeutic agents, and the need for standardized detection methods for Piezo channel-based diagnostic biomarkers. In-depth investigation of these aspects will advance the application of Piezo channels in cancer diagnosis and treatment.

## Data Availability

Not applicable.
